# Long-term exposure to air pollution and hospitalization for dementia in the Rome longitudinal study

**DOI:** 10.1186/s12940-019-0511-5

**Published:** 2019-08-09

**Authors:** Francesco Cerza, Matteo Renzi, Claudio Gariazzo, Marina Davoli, Paola Michelozzi, Francesco Forastiere, Giulia Cesaroni

**Affiliations:** 1 Department of Epidemiology- Lazio Regional Health Service, ASL Roma 1, Rome, Italy; 2 Department of Occupational & Environmental Medicine, INAIL, Monteporzio Catone, RM Italy; 3 National Research Council-IBIM, Palermo, Italy; 4 Environmental Research Group, King’s College, London, UK

**Keywords:** Dementia, Air pollution, Cohort analysis, Rome longitudinal study, Alzheimer’s disease, Vascular dementia

## Abstract

**Background:**

Few studies have explored the role of air pollution in neurodegenerative processes, especially various types of dementia. Our aim was to evaluate the association between long-term exposure to air pollution and first hospitalization for dementia subtypes in a large administrative cohort.

**Methods:**

We selected 350,844 subjects (free of dementia) aged 65–100 years at inclusion (21/10/2001) and followed them until 31/12/2013. We selected all subjects hospitalized for the first time with primary or secondary diagnoses of various forms of dementia. We estimated the exposure at residence using land use regression models for nitrogen oxides (NOx, NO_2_) and particulate matter (PM) and a chemical transport model for ozone (O_3_). We used Cox models to estimate the association between exposure and first hospitalization for dementia and its subtypes: vascular dementia (Vd), Alzheimer’s disease (Ad) and senile dementia (Sd).

**Results:**

We selected 21,548 first hospitalizations for dementia (7497 for Vd, 7669 for Ad and 7833 for Sd). Overall, we observed a negative association between exposure to NO_2_ (10 μg/m^3^) and dementia hospitalizations (HR = 0.97; 95% CI: 0.96–0.99) and a positive association between exposure to O_3_, NOx and dementia hospitalizations, (O_3_: HR = 1.06; 95% CI: 1.04–1.09 per 10 μg/m^3^; NOx: HR = 1.01; 95% CI: 1.00–1.02 per 20 μg/m^3^).H. Exposure to NOx, NO_2_, PM_2.5_, and PM_10_ was positively associated with Vd and negatively associated with Ad. Hospitalization for Sd was positively associated with exposure to O_3_ (HR = 1.20; 95% CI: 1.15–1.24 per 10 μg/m^3^).

**Conclusions:**

Our results showed a positive association between exposure to NOx and O_3_ and hospitalization for dementia and a negative association between NO_2_ exposure and hospitalization for dementia. In the analysis by subtype, exposure to each pollutants (except O_3_) demonstrated a positive association with vascular dementia, while O_3_ exposure was associated with senile dementia. The results regarding vascular dementia are a clear indication that the brain effects of air pollution are linked with vascular damage.

**Electronic supplementary material:**

The online version of this article (10.1186/s12940-019-0511-5) contains supplementary material, which is available to authorized users.

## Background

The estimated number of people living with dementia worldwide in 2015 was 46.8 million. This number will almost double every 20 years, resulting in problems for individuals, families, and society [1]. Among the several types of dementia, Alzheimer’s disease is the most common and accounts for an estimated 60 to 80% of cases [2], whereas Vascular dementia, caused by cerebrovascular diseases, is the second most common form of dementia, accounting for 10 to 20% of cases [3]. Dementia is a syndrome characterized by a cognitive and functional decline. It is usually preceded by mild cognitive impairment, and generally, it is difficult to distinguish between the two conditions [4]. Dementia commonly occurs later in life as a consequence of the cerebrovascular and neurodegenerative processes that begin earlier in life [5]. Alzheimer’s disease is characterized by a progression in cognitive decline and in ability to function. It is often characterised also by behavioural disturbances, which tend to become more frequent as the severity of dementia increases. People can lose their independence, becoming unable to take care of themselves. Although there can be plateaus during the illness, the decline tends to accelerate and increase [6]. Vascular dementia can manifest soon after a vascular event that can cause a sudden deterioration in cognitive function, or can manifest slowly after multiple cerebral infarctions or diffuse white matter disease. The course of vascular dementia is less predictable than Alzheimer’s disease, since if underlying vascular disease can be stabilised, a relative stability may be seen for a period [3, 6].

Age is the most important known risk factor for dementia [2], and given the increasing elderly population, dementia will represent one of the most relevant public health issues. Diabetes [7, 8], mid-life obesity [9, 10], smoking [11, 12], brain injury [13], and depression [14, 15] are associated with a higher dementia risk [16]. There are also factors associated with a decreased risk of dementia, including physical activity [17, 18] and years of formal education [16, 19]. However, the aetiology of dementia is not fully understood, because genetic factors [20] and the most common risk factors do not fully explain dementia risk [21, 22]. Taking into account of non-independence between potentially modifiable risk factors, Norton and colleagues estimated that one third of Alzheimer’s disease cases might be attributable to education, physical inactivity, hypertension, diabetes, obesity, depression, and smoking [21].

The role of long-term exposure to air pollution in the onset of dementia is still controversial. In last decade, there has been increased interest in the effects of air pollution on the central nervous system and neurodegeneration. According to both epidemiological and toxicological studies, exposure to air pollution, particularly particulate matter, seems to be associated with decreased cognitive function [23–27]. Potential biological pathways, such as systemic inflammation, have been highlighted in order to explain this relationship [25, 27]. In particular, it is recognized that older brains are more vulnerable to pro-inflammatory stimuli, and one of the possible mechanisms of air pollution’s adverse effects on brain health is oxidative stress [25, 27]. Few studies have analysed the role of air pollution exposure in the development of dementia, using different study designs and different measures of exposure, and showed an association between air pollution exposure and dementia [28–34]. Only two studies differentiated between types of dementia, studying Alzheimer’s disease and vascular dementia [28, 32]. The Swedish cohort study found similar results by dementia types [28], while the case control study by Wu and colleagues found stronger associations between O_3_ and PM_10_ and Alzheimer’s disease compared to vascular dementia [32].

The aim of the present study was to evaluate the association between air pollution exposure (PM_10_, coarse PM (particles with a size fraction between 2.5 and 10 μm), PM_2.5_, PM_2.5_ absorbance, NO_2_, NOx, summer O_3_, and distance to high-traffic roads) and first hospitalization for dementia in a large cohort of residents in Rome [35, 36]. Furthermore, we separately investigated the effect of air pollution on hospitalization for three types of dementia: vascular dementia, Alzheimer’s disease and senile dementia.

## Methods

### Study population

We defined the study population from the Rome Longitudinal Study, an administrative cohort [35, 36] that included all residents in Rome who filled out the questionnaire of the population census from 2001 on October 21st, 2001 and followed them using administrative data. The Rome Longitudinal Study included all subjects who had resided in Rome for at least 5 years and were not living in institutions (prisons, hospitals, or nursing homes) at the time of the census. For this study, we selected all subjects aged 65 years or older at inclusion and followed them until December 31st, 2013 or until 100 years of age, death, migration or hospitalization for dementia, whichever came first.

Individual information, recorded at the 2001 census, was available, including gender, age, educational level, occupational status, marital status, and place of birth. Residential history and vital status were available from the Municipal Register data.

### Study outcomes

We used data from the Hospital Discharge Registry (HDR), included in the Regional Health Information System (HIS), which covers both public and private health care providers and collects up to six diagnoses and procedure codes (ICD-9-CM) for each hospital discharge.

We used the HDR to identify dementia hospitalizations from October 21st, 2001 to December 31st, 2013. We selected from the HDR subjects hospitalized for the first time during the follow-up in the Lazio Region with a primary or a secondary diagnosis of Jakob-Creutzfeldt disease (ICD9-CM: 046.1), senile dementia (ICD9-CM: 290.0, 290.2, 290.3), presenile dementia (ICD9-CM: 290.1), vascular dementia (ICD9-CM: 290.4), persistent mental disorders due to conditions classified elsewhere (ICD9-CM: 294), Alzheimer’s disease (ICD9-CM: 331.0), Pick's disease (ICD9-CM: 331.1), or Dementia with Lewy bodies (ICD9-CM: 331.82). We studied first dementia hospitalizations overall (including all the mentioned codes) and for subtypes: vascular dementia (ICD9-CM: 290.4), Alzheimer’s disease (ICD9-CM: 331.0), and senile dementia (ICD9-CM: 290.0, 290.2, 290.3), which were the most frequent dementia codes in discharge records. The codes for senile dementia are likely used for elderly when a more specific diagnosis is difficult to be determined.

To study the first hospitalization for dementia and dementia subtype, we excluded from the analyses the subjects hospitalized for dementia before the beginning of the follow-up (from January 1st, 1996 to October 21st, 2001).

### Exposure assessment

To assess environmental exposure at the residential address at the time of inclusion, we used the Land Use Regression (LUR) models developed within the European Study of Cohorts for Air Pollution Effects (ESCAPE project) for the city of Rome. The pollutants of interest were particulate matter (size fraction < 10 μm: PM_10_, < 2.5 μm: PM_2.5_, soot (PM_2.5_ absorbance), and 2.5–10 μm: coarse PM), and nitrogen oxides (NOx and NO_2_) [37, 38]. The models’ R^2^ values ranged from 71% (PM_2.5_) to 86% (NO_2_), and the cross-validated R^2^ values ranged from 59% (PM_10_) to 79% (PM_2.5_ absorbance). Details on the models and measurement campaigns can be found elsewhere [37–40]. Briefly, particulate matter was measured in 20 sites, and nitrogen oxides were measured in 40 sites in three two-week periods during 2010. Measures were averaged using a continuous background monitoring site operating for the entire year in order to estimate an annual average measure of the concentration of pollutants at each site. To predict the concentration of pollutants, several traffic and land use variables were used. Following a strict protocol based on a multilinear regression model, an equation for each pollutant was identified. The produced equations were applied to estimate the pollutant concentration at each individual address.

To estimate summer (from May to September) daily ozone (8 h) exposure, we used the Flexible Air quality Regional Model (FARM), a chemical transport model. The FARM is a three-dimensional Eulerian model used to simulate the transport and multiphase chemistry of pollutants in the atmosphere. This model was applied over the city of Rome with a grid resolution of 1 × 1 km, using emission inventory data, modelled meteorology and measurements taken in 2005 [41].

Furthermore, as a proxy measure of exposure to traffic, we analysed the distance between residential addresses and the nearest high-traffic road (HTR; roads with > 10,000 vehicles per day) [36]. Distance (m) was measured using ArcGIS software.

### Statistical analysis

We calculated standardized incidence rates for the first hospitalization for dementia using four age groups (65–74, 75–84, 85+) and the Italian population in 2008 as the standard population. We used the Cox proportional hazards models, with age as the time scale, to estimate the association between long-term air pollution exposure (PM_10_, coarse PM, PM_2_._5_, PM_2.5_ absorbance, NO_2_, NOx, and summer O_3_) and first hospitalization for dementia. To explore the effects of air pollution on the subtypes of dementia, we conducted a separate analysis for the first hospitalization for each classification: vascular dementia, Alzheimer’s disease and senile dementia (a subject could have been classified as having more than one type).

We considered several variables as possible confounders or effect modifiers: age, sex, place of birth (Rome or other), marital status (married, single, separated/divorced or widowed), and educational level (primary school or less, junior high school, high school or university). We used a small area (census block, average 500 inhabitants) composite index of socioeconomic position (SEP, five levels: from very high to very low) that allowed us to better characterize residential deprivation [42]. We considered as potential confounders the hospital discharges occurred before the inclusion for diabetes, brain injuries and, since we did not have information on smoking habit, the discharges for chronic obstructive pulmonary disease (see Additional file 1 with ICD-9-CM codes for selection).

We stratified the baseline hazard function by sex, which was the only variable that did not satisfy the proportional-hazards assumption (tested by Schoenfeld’s residuals). Air pollutants were included as continuous variables. We expressed hazard ratios (HR) and relative 95% confidence intervals per fixed increases of each pollutant: 10 μg/m^3^ for PM_10_, NO_2_ and Summer O_3_, 5 μg/m^3^ for PM_2.5_ and coarse PM, 20 μg/m^3^ for NOx and 1 unit (10^− 5^/m) for PM_2.5_ absorbance. We defined five categories of distance to an HTR: less than 50 m, 50–100 m, 101–200 m, 201–300 m, and more than 300 m from an HTR. In order to address possible confounding between pollutants, for the first hospitalization for dementia we performed two pollutants models, when the correlation was < 0.70 and the main analyses showed statistical significant results.

We evaluated a possible effect modification adding an interaction term between the exposures and the effect modifiers (sex, age, SEP, or education), and we performed the likelihood ratio test to compare the goodness of fit of the models with and without the interaction term.

To account for the possible bias due to deaths that occurred before dementia onset, we performed a competing risks analysis using the Fine and Grey method, where we considered death as a competing event [43]. Only study participants with no missing information from any of the exposures and confounders in the main model were included in analyses.

Finally, to explore possible misclassification, we performed additional sensitivity analyses. We selected narrower subgroups of cases. The first subgroup was composed by the first of at least two hospitalizations of the same subtype. The second subgroup included hospitalizations with only primary diagnosis of dementia and subtypes. To explore the possible misclassification of exposure we performed the analysis on subjects who did not change residence during the entire follow-up.

Analyses were performed using STATA13 (StataCorp. 2013. Stata Statistical Software: Release 13. College Station, TX: StataCorp LP).

## Results

After the exclusion of prevalent cases of dementia (*N* = 4292; 1%) and subjects with missing exposure information (*N* = 4417; 1%), we selected 350,844 subjects.

Table 1 shows the characteristics of the study population at the beginning of the follow-up and first hospitalization for dementia. Our study population was composed of 145,994 men (42%) and 204,900 women (58%). The mean age at inclusion was 74.5 years (standard deviation (SD) 6.9). A proportion of 51% of the population had a primary school or less education, 58% were married, and 33% were born in Rome. During the follow-up (10.6 years), 21,548 subjects were hospitalized for the first time for dementia (7916 men and 13,632 women). The standardized hospitalization rates were higher in women, in those living without a partner, in less educated people and in subjects with a very low or low socioeconomic position.

**Table 1:** Baseline characteristics of the study population and first hospitalization for dementia

Characteristics	Population		First hospitalization for dementia		
	N	%	N	%	SHR^a^ (10,000 P-Y)
Total	350,844	100	21,548	100	88
Age class					
65–69	110,334	31.5	3214	14.9	
70–74	95,602	27.3	5367	24.9	
75–79	73,421	20.9	6332	29.4	
80–84	38,620	11.0	3883	18.0	
85–89	23,299	6.6	2116	9.8	
90+	9568	2.7	636	3.0	
Age mean (SD)	74.5 (6.8)		77.1 (6.5)		
Gender					
Men	145,944	41.6	7916	36.7	85
Women	204,900	58.4	13,632	63.3	90
Place of Birth ^b^					
Rome	114,112	32.5	6685	31.0	92
Other	236,732	67.5	14,863	69.0	87
Marital Status					
Married	202,004	57.6	10,787	50.1	83
Single	24,626	7.0	1585	7.4	91
Separated	12,043	3.4	642	3.0	97
Widowed	112,171	32.0	8534	39.6	98
Education ^b^					
Primary School or less	178,062	50.8	12,423	57.7	100
Junior high School	72,823	20.8	4146	19.2	85
High School	60,861	17.4	3044	14.1	69
University	39,098	11.1	1935	9.0	72
Area-based SEP^c^					
Very High	75,772	21.6	4271	19.8	78
High	75,595	21.6	4556	21.1	84
Medium	72,325	20.6	4459	20.7	89
Low	68,080	19.4	4321	20.1	96
Very Low	59,072	16.8	3941	18.3	101
CVD prevalent					
*Yes*	89,054	25.4	6650	30,9	113
*No*	261,790	74.6	14,898	69,1	80
Stroke prevalent					
Yes	6560	1.9	638	3.0	168
No	344,284	98.1	20,910	97.0	87
COPD					
Yes	19,472	94.5	1402	6.5	123
No	331,372	5.6	20,146	93.5	87
Brain injury					
Yes	2257	0.6	194	0.9	121
No	348,587	99.4	21,354	99.1	88
Distance to HTR (m)					
< 50	67,515	19.2	4270	19.8	90
50–100	53,729	15.3	3286	15.3	87
101–200	98,262	28.0	6066	28.2	88
201–300	56,292	16.0	3445	16.0	87
> 300	75,046	21.4	4481	20.8	88

^a^ *SHR* Standardized hospitalization rate, standardized by sex and age (age groups: 65–69; 70–74; 75–79; 80–84; 85–89; 90+) considering Italian residents in 2008 as standard population (source:www.demo.istat.it)

^b^ Information at 2001 census of the population

^c^ *SEP* Socioeconomic position; Information at baseline residence

Table 2 shows the characteristics of the first hospitalization for different types of dementia considered. We selected 7497 first time hospitalized people for vascular dementia (3083 men and 4414 women), 7669 for Alzheimer’s disease (2679 men and 4990 women) and 7833 for senile dementia (2654 men and 5179 women). The mean age at the baseline was approximately 76 years (SD 6) for subjects who developed Alzheimer’s disease, whereas it was 78 (SD 7) for subjects who developed vascular or senile dementia. The standardized hospitalization rate was 24 per 10,000 person-years for each types of dementia. The distribution of this rate was similar in all types of dementia by place of birth, educational level and socio-economic position. Higher rates were observed in widowed and single subjects for vascular and senile dementia and in married and widowed subjects for Alzheimer’s disease.

**Table 2:** Incidence of first hospitalization for vascular dementia, Alzheimer’s disease and senile dementia

Characteristics	Vascular dementia			Alzheimer’s disease			Senile dementia		
	First hospitalization		SHR ^a^	First hospitalization		SHR ^a^	First hospitalization		SHR ^a^
	N	%	(10,000 P-Y)	N	%	(10,000 P-Y)	N	%	(10,000 P-Y)
Total	7497	100	32	7669	100	28	7833	100	32
Age									
65–69	983	13.1		1347	17.6		1033	13.2	
70–74	1721	23.0		2244	29.3		1806	23.1	
75–79	2185	29.1		2319	30.2		2295	29.3	
80–84	1507	20.1		1182	15.4		1490	19.0	
85–89	837	11.2		493	6.4		907	11.6	
90+	264	3.5		84	1.1		302	3.9	
Age mean (SD)	77.7 (6.5)			75.8 (5.8)			77.7 (6.6)		
Gender									
Men	3083	41.1	35	2679	34.9	25	2654	33.9	29
Women	4414	58.9	30	4990	65.1	30	5179	66.1	35
Place of Birth ^b^									
Rome	2326	31.0	34	2336	30.5	28	2453	31.3	35
Other	5171	69.0	31	5333	69.5	28	5380	68.7	32
Marital Status ^b^									
Married	3690	49.2	29	4200	54.8	28	3629	46.3	30
Single	603	8.0	36	416	5.4	21	649	8.3	37
Separated	227	3.0	42	206	2.7	25	265	3.4	37
Widowed	2977	39.7	36	2847	37.1	29	3290	42	37
Education ^b^									
Primary School	4445	59.3	37	4445	58.0	32	4475	57.1	36
Junior high School	1452	19.4	31	1483	19.3	27	1463	18.7	31
High School	970	12.9	23	1087	14.2	22	1193	15.2	28
University	630	8.4	24	654	8.5	22	702	9	28
Area-based SEP ^c^									
Very High	1355	18.1	25	1510	19.7	25	1605	20.5	30
High	1579	21.0	30	1605	20.9	27	1714	21.9	32
Medium	1639	21.9	34	1537	20.0	28	1578	20.1	32
Low	1552	20.7	36	1565	20.4	30	1512	19.3	35
Very Low	1372	18.3	38	1452	18.9	32	1424	18.2	38
CVD prevalent									
Yes	2722	36.3	40	2046	26.7	32	2354	30.1	41
No	4775	63.7	19	5623	73.3	27	5479	69.9	30
Stroke prevalent									
Yes	354	4.7	87	115	1.5	29	193	2.5	50
No	7143	95.3	23	7554	98.5	28	7640	97.5	33
COPD									
Yes	583	7.8	51	392	5.1	33	526	6.7	47
No	6914	92.2	31	7277	94.9	28	7307	93.3	32
Brain injury									
Yes	75	1.0	46	62	0.8	37	71	0.9	44
No	7422	99.0	32	7607	99.2	28	7762	99.1	33
Distance to HTR (m)									
< 50	1583	21.1	26	1446	18.9	27	1516	19.4	33
50–100	1201	16.0	25	1149	15.0	27	1184	15.1	32
101–200	2154	28.7	32	2173	28.3	29	2108	26.9	31
201–300	1122	15.0	30	1253	16.3	28	1293	16.5	33
> 300	1437	19.1	29	1648	21.5	29	1732	22.1	37

^a^ *SHR* Standardized hospitalization rate, standardized by sex and age (age groups: 65–69; 70–74; 75–79; 80–84; 85–89; 90+) considering Italian residents in 2008 as standard population (source:www.demo.istat.it)

^b^ Information at 2001 census of the population

^c^ *SEP* Socioeconomic position; Information at baseline residence

Table 3 shows the average exposure levels of the population (mean (SD and interquartile range)) at inclusion, which were 36.9 μg/m^3^ (5.3 and 4.9) for PM_10_, 17.4 μg/m^3^ (3.4 and 1.8) for coarse PM, 19.7 μg/m^3^ (2.0 and 3.4) for PM_2.5_, 2.76 10^− 5^/m (0.48 and 0.33) for PM_2.5_ absorbance, 43.9 μg/m^3^ (10.3 and 12.7) for NO_2_, 87.4 μg/m^3^ (24.1 and 32.6) for NOx, and 97.6 μg/m^3^ (5.9 and 4.6) for O_3_.

**Table 3:** Average annual concentrations of air pollutants and distance to HTR at baseline address

Characteristics	Mean ± SD	Min	P25	P50	P75	Max	IQR
PM_10_ (μgm^3^)	36.9 ± 5.3	29.6	33.5	35.4	38.4	58.2	4.9
PM_2.5_ (μg/m^3^)	19.7 ± 2.0	17.0	18.5	19.2	20.3	27.4	1.8
Coarse PM (μg/m^3^)	17.4 ± 3.4	9.8	15.2	16.8	18.6	31.4	3.4
PM_2.5_ absorbance (10^−5^/m)	2.76 ± 0.48	2.16	2.49	2.66	2.83	4.77	0.33
NO_2_ (μg/m^3^)	43.9 ± 10.3	13.2	36.8	43.3	49.5	84.9	12.7
NOx (μg/m^3^)	87.4 ± 24.1	16.9	70.0	85.4	102.6	173.4	32.6
O_3_ (μg/m^3^)	97.6 ± 5.9	54.6	95.7	98.8	100.3	112.8	4.6
Distance to HTR (m)	203 ± 198	2	71	149	272	946	201

*HTR* High-Traffic Road

EU air quality standards for annual averages are 25 μg/m3 for PM2.5, 40 μg/m3 for PM10 and NO2. EU target value for maximum daily 8-h mean O3 is 120 μg/m3

The average distance to an HTR was 203 m (198 and 201). We found a relatively high correlation between particulate matter and nitrogen oxides (ranging from 0.52 [NOx - PM_2.5_ absorbance] to 0.71 [coarse PM - NO_2_]), likely due to similar emission sources, while the correlation was lower between O_3_ and other pollutants (ranging from − 0.12 [NOx - O_3_] and − 0.02 [PM2.5 - O_3_]) (see Additional file 2).

Table 4 shows the association (adjusted Hazard ratios, HRs) between long-term exposure to air pollution and first hospitalization for dementia, vascular dementia, Alzheimer’s disease and senile dementia. Unadjusted HRs are presented in Additional file 3. Overall, we found a negative association between long-term exposure to NO_2_ and dementia (HR = 0.98, 95% CI: 0.96, 0.99 per 10 μg/m^3^ increases) and a positive association between exposure to O_3_ and dementia (HR = 1.06, 95% CI: 1.03, 1.08 per 10 μg/m^3^ increases). The associations of dementia with coarse PM and NOx exposures were negative and positive, respectively (HR = 0.98, 95% CI: 0.96, 1.00 per an increase of 5 μg/m^3^ of coarse PM and HR = 1.01, 95% CI: 1.00, 1.02 per an increase of 20 μg/m^3^ of NOx). There was no evidence of associations between hospitalization for dementia and exposure to the other pollutants nor to distance to HTR.

**Table 4:** Association between long-term exposure to air pollution and overall dementia and dementia subtypes

Characteristics	Dementia			Vascular dementia			Alzheimer’s disease			Senile dementia		
	*N* = 21,548			*N* = 7500			*N* = 7671			*N* = 7835		
	HR ^a^	95%CI		HR ^a^	95%CI		HR ^a^	95%CI		HR ^a^	95%CI	
PM_10_ (10 μg/m^3^)	1.00	0.98	1.03	1.06	1.02	1.10	0.95	0.91	0.99	0.98	0.94	1.02
Coarse PM(5 μg/m^3^)	0.98	0.96	1.00	1.06	1.03	1.09	0.91	0.87	0.94	0.97	0.93	1.00
PM_2.5_ (5 μg/m^3^)	0.99	0.96	1.02	1.07	1.01	1.12	0.91	0.85	0.97	0.98	0.92	1.03
PM_2.5_ abs (10^−5^/m)	1.00	0.98	1.03	1.15	1.10	1.19	0.91	0.86	0.96	0.93	0.88	0.98
NO_2_ (10 μg/m^3^)	0.97	0.96	0.99	1.05	1.03	1.07	0.91	0.89	0.94	0.96	0.94	0.98
NOx (20 μg/m^3^)	1.01	1.00	1.02	1.08	1.06	1.10	0.96	0.94	0.98	0.99	0.97	1.01
O_3_ (10μg/m^3^)	1.06	1.03	1.08	1.02	0.98	1.06	0.98	0.95	1.02	1.19	1.15	1.24
Distance to HTR (m)												
*< 50*	1.01	0.97	1.06	1.17	1.10	1.24	0.97	0.90	1.04	0.90	0.83	0.97
*50–100*	0.98	0.93	1.02	1.11	1.03	1.19	0.96	0.89	1.04	0.88	0.80	0.95
*101–200*	0.99	0.95	1.03	1.10	1.03	1.17	0.99	0.92	1.05	0.87	0.80	0.93
*201–300*	1.00	0.95	1.04	1.02	0.94	1.09	1.00	0.93	1.08	0.94	0.87	1.02
*> 300*	1.00	ref.		1.00	ref.		1.00	ref.		1.00	ref.	
*p-trend* ^*b*^	0.827			< 0.001			0.206			0.001		

^a^ Models adjusted for age, education, place of birth, marital status, area-based socioeconomic position with baseline hazard function stratified by sex

^b^ Wald test across categories of exposure

We found an overall statistically significant positive association between first hospitalization for vascular dementia and each pollutant (with the exception of O_3_) and distance to HTR. In contrast, for Alzheimer’s disease, opposite results emerged. We found a negative effect of PM_2.5_ absorbance, NO_2_, and distance to an HTR, and a positive effect of O_3_ (HR = 1.20, 95% CI: 1.15, 1.24 per an increase of 10 μg/m^3^) on senile dementia.

The association between O_3_ and dementia remained when we adjusted for other pollutants in two-pollutant models (e.g. adjusted for NO_2_: HR = 1.05, 95% CI: 1.03, 1.08, for 10 μg/m^3^ increases of O_3_). The association did not change when we studied the exposure of NO_2_ and NOx adjusted for other pollutants.

We saw no effect modification by age, sex, level of education or SEP (data not shown) for dementia outcome. We observed a higher risk in women compared to men for increased exposure to NOx for vascular dementia (HR = 1.06, 95% CI: 1.03, 1.09 per an increase of 20 μg/m^3^ in men; HR = 1.10, 95% CI: 1.07, 1.12 per an increase of 20 μg/m^3^ in women; *p*-value for interaction = 0.053). We also found an effect modification by SEP, with a higher risk of senile dementia in lower socioeconomic positions for increased exposure to O_3_. Per an increase of 10 μg/m^3^ O_3_, we observed a HR = 1.27 (95% CI: 1.18, 1.36) in the very low socioeconomic group and a HR = 1.08 (95% CI: 0.98, 1.18) in the very high socioeconomic group (p-value for interaction = 0.047).

The negative association between nitrogen dioxide and overall dementia hospitalization could not be explained by comorbidities such as COPD, diabetes, or brain injuries, nor by mortality as competing risk, nor by the case definition, nor by changes of residence during the follow-up (Additional file 4).

The sensitivity analyses did not show different results for vascular dementia, Alzheimer and senile first hospitalization (Additional files 5, 6 and 7).

## Discussion

We observed a positive association between residential exposures to NOx and O_3_ and first hospitalization for dementia, an inverse association with NO_2_ and dementia, and no associations with other pollutants. Among the first hospitalization by dementia subtype, we found strong association between air pollution exposure and vascular dementia, and not clear results for Alzheimer’s disease and senile dementia. The results were confirmed by sensitivity analyses performed on the subgroup of subjects with narrower case definitions and in analyses including death as the competing event.

Only recently, seven studies have explored the role of air pollution exposure on dementia (see Additional file 8). Six were cohort studies, including five studies on disease incidence and one on hospital admissions in the United States [28–31, 34, 44]. Our results regarding the relationship between residential exposure to air pollution and hospitalization for dementia are inconsistent with previous reports [28, 29, 34]. This could depend mainly by the different outcomes chosen; most of the previous studies analysed the incidence of the disease as the outcome, whereas we studied the first hospitalization. The access to hospital does not represent the onset of neurodegeneration; it partly represents the severity of neurocognitive disorders, and partly the severity of important comorbid conditions, that require a hospital admission. Our findings are in contrast with the only study on hospital admissions for dementia [44]. We found null results for particulate matter, while the hazard ratio reported in the US was 1.08 (1.05–1.11) per 1 μg/m^3^ increase in PM_2.5_ [44]. However, in their study, Kioumourtzoglou and colleagues analysed data from 50 cities and found some geographical variability in their results [44].

To our knowledge, only a Canadian study investigated the association of exposure to O_3_ on overall dementia and showed an absence of association [34], while in our data we found a 6% higher hospitalization rate per 10 μg/m^3^ in summer ozone exposure.

The findings on vascular dementia, with a strong association with all pollutants (except O_3_ but including traffic exposure) are comparable to those reported in the literature. A case-control study in Taiwan showed a strong positive significant association with PM_10_ [32], and a Swedish cohort study including 1806 adults showed a positive effect of NOx exposure [28]. In contrast to Wu and colleagues, we did not find any association between ozone exposure and vascular dementia, but we found higher hospitalization risks with the increase of all traffic-related pollutants (PM_2.5_ absorbance, NOx, NO_2_ and decreasing distance to high-traffic roads). Vascular dementia is characterized by a different pathogenesis in respect to other dementias, such as Alzheimer’s disease. Vascular damages triggered by large vessel atherosclerosis and small vessel arteriosclerosis cause cortical and subcortical infarcts, sub-infarct ischaemic lesions and large and small cerebral haemorrhages that are responsible for the onset of vascular dementia [45]. Moreover, it is well established that long-term exposure to air pollution is linked to atherosclerosis and vascular diseases [46]. Despite the fact that vascular dementia patients share similar neurological symptoms with subjects affected by other types of dementia, they present different subclinical conditions as described above. Those different pathogenesis mechanisms could explain our results. In fact, patients affected by vascular dementia should receive different treatment, provided only by hospitals, compared to Alzheimer’s patients who are usually treated at home or in nursing facilities (as described in detail below).

We found statistically significant negative associations between Alzheimer’s disease and all pollutants, with the exception of O_3._ These results contrast with previous reports: Kioumourtzoglou et al. reported a HR = 1.15 (1.11, 1.19) per 1 μg/m^3^ increase in PM_2.5_, Oudin et al. found a HR = 1.02 (0.92, 1.14) per 10 μg/m^3^ increase in NOx_,_ and the case control study showed an association between both ozone and PM_10_ and Alzheimer’s disease [28, 44]. However, independently of previous research, our results have important limitations. In contrast to other cohort studies on the incidence of dementia, we analysed hospital admissions as the outcome, which do not reflect the onset of the disease, nor the gravity, but indicates access to services. Alzheimer’s disease and dementia are not conditions that systematically require hospital admissions. In Italy, they are usually treated at home, with a large burden on familial components, and at a later stage in nursing homes.

To our knowledge, there are no other studies that have investigated the possible role of air pollution in senile dementia. Similar to Alzheimer’s disease, we found negative associations with all traffic-related pollutants (soot, NO_2_, NOx) and closer distances to high traffic roads. One possible explanation could rely on the same hypothesis of the Alzheimer’s results. Despite the fact that some mechanisms of the onset of senile dementia are still unknown, these diseases share the same risk factors and subjects could present similar clinical conditions. We found a strong association between long-term O_3_ exposure and hospitalizations for senile dementia, with a 20% higher risk of being hospitalized per 10 μg/m^3^ increase in summer ozone. Socioeconomic position was an effect modifier in the association between ozone and hospital admissions for senile dementia, with a higher risk in disadvantaged groups compared to subjects with high socioeconomic levels. The differences we found between socioeconomic levels can be attributed to behavioural changes arising from adaptive strategies [47].

During the last years, several epidemiological and toxicological studies highlighted the potential impact of air pollution on the central nervous system. The pathways involved in brain responses are those identified for responses in the circulatory system, as stated by Block and colleagues (2012): “(1) the release of inflammatory and oxidative stress mediators by the lungs into the systemic circulation; (2) interactions between pollutants and pulmonary neuronal afferents resulting in autonomic imbalance; and (3) direct translocation of particle constituents or particles from the respiratory tract into the systemic circulation” [25]. The role of particulate matter and nitrogen oxides in cardiovascular disease is well-established [48–50], hence the association we found between air pollution exposure and vascular dementia is not surprising. The evidence of long-term exposure to ozone has been less studied compared to other pollutants. However, ozone activates pro-inflammatory genes [51] Both acute and long-term impacts of ozone on mortality have been reported in humans [52, 53], as the adverse neurobehavioral effects in adults, reducing coding ability, attention and short-term memory, characteristics common to all types of dementia [54].

This is the largest population-based cohort study in Europe investigating the role of long-term exposure to air pollution in dementia and to our knowledge, no other studies have investigated senile dementia in particular. The exposure was estimated at residential addresses, with the exception of ozone where a 1 km^2^ area was used for all subjects, and several individuals’ information was available. The study size allowed us to carry out analysis by type of dementia, whereas in the literature, studies generally focus either on overall dementia or on Alzheimer’s disease.

The main limitation of this analysis is the use of dementia hospitalizations as outcome, with a possible misclassification of cases. Generally, the identification of dementia cases is based on different sources ranging from hospital discharge registry to drug registry or neurological reports. In our study, data from drug registries were not available for the entire period of interest, thus we limited case collection to only hospital discharge registries. The possible misclassification is due to the fact that subjects who are hospitalized for dementia are not representative of all dementia cases. Moreover, the use of hospitalization data could introduce a selection bias by dementia subtype. In fact, a vascular dementia case, which probably had a previous episode of stroke or other important cardiovascular diseases, can be more likely to be hospitalized and selected than an Alzheimer’s or a senile case. In order to distort the results the selection should be related to air pollution exposure, but particularly the results on dementia subtype need to be interpreted cautiously, because they could depend on the coding accuracy of secondary diagnoses.

Our cohort was based on administrative data, and information on many relevant individual risk factors, such as genetic factors, BMI, physical activity, and smoking, were not available. We addressed these issues by adjusting for a small area composite index of socioeconomic position that is associated with lifestyle behaviours, thus making confounding less probable, but residual confounding cannot be excluded [55]. Furthermore, we excluded from the analysis subjects without exposure assessment and considered loss to follow-up those who migrated from Rome during the study period. Since the characteristics of excluded or migrated individuals were similar to those included in the analysis, a bias was probably not introduced.

Another strong limitation was exposure assessment. The land use regression models were based on air pollution measurements taken in 2010 and applied to residential addresses at the beginning of the follow-up. Elsewhere, it has been demonstrated that the spatial distribution of nitrogen dioxide concentrations did not change in Rome over time [56], but we need to make the same assumption for the remaining pollutants. In Rome, data from fixed monitors show a decline of pollutants concentrations only in traffic sites during the follow-up. This decrease combined to the implementation of the traffic-limited zone in the historical city centre, might have improved air quality in the urban core.

Finally, air pollution concentrations in Rome are not evenly distributed [35]. The city centre, characterized by high socioeconomic positions and a large percentage of elderly residents, is the most polluted area of Rome. Well-off families, which are the most exposed, have the means of treating their relatives at home. Moreover, socioeconomic inequalities in hospitalizations have been reported, and high socioeconomic class subjects are less likely to need all-causes hospitalizations than those in deprived groups of the population [57]. Thus, even if we adjusted the models for level of education and socioeconomic position, possibly the negative association we found could be attributable to residual confounding for socioeconomic status and an indication of a differential use of health services.

## Conclusions

We found a positive association between residential exposure to NOx and ozone with first hospitalization for dementia in Rome. Exposures to particulate matter and nitrogen oxides were associated with hospitalizations for vascular dementia. We could not find a reasonable explanation for the negative associations we found in Alzheimer’s disease, and the results need to be interpreted cautiously, because on one side residual confounding of unmeasured lifestyle risk factors could play an important role, and on the other side using hospitalizations to identify dementia cases could lead to possible misclassification of cases.

Air pollution is pervasive, global, and harmful for health. Further efforts to reduce exposure in big cities are needed and have also the potential for beneficial effects on neurological health in elderly.

## Additional files

Additional file 1: ICD9-CM codes for comorbid conditions. (DOCX 13 kb)

Additional file 2: Pearson correlation coefficients of exposure to air pollutants. (DOCX 14 kb)

Additional file 3: Association between long-term exposure to air pollution and dementia, vascular dementia, Alzheimer disease and senile dementia. Rome 2001–2013. (DOCX 16 kb)

Additional file 4: Association between long-term exposure to air pollution and first hospitalization for dementia. Sensitivity analyses, Rome 2001–2013. (DOCX 16 kb)

Additional file 5: Association between long-term exposure to air pollution and first hospitalization for vascular dementia. Sensitivity analyses, Rome 2001–2013. (DOCX 16 kb)

Additional file 6: Association between long-term exposure to air pollution and first hospitalization for Alzheimer’s disease. Sensitivity analyses, Rome 2001–2013. (DOCX 16 kb)

Additional file 7: Association between long-term exposure to air pollution and first hospitalization for senile dementia. Sensitivity analyses, Rome 2001–2013. (DOCX 20 kb)

Additional file 8: Previous studies on Dementia disease (overall dementia, Alzheimer’s disease and vascular dementia) and air pollution. (DOCX 16 kb)

## Data Availability

Not applicable
