# Supplementary material for: Long-term exposure to air pollution and hospitalization for dementia in the Rome longitudinal study
Source: Environ Health. 2019 Aug 9;18:72. doi: 10.1186/s12940-019-0511-5 (PMC6689157; doi:10.1186/s12940-019-0511-5)
Supplement: Supplementary file 1 — ICD9-CM codes for comorbid conditions. (DOCX 13 kb) [file 12940_2019_511_MOESM1_ESM.docx]

**Additional file 1.** ICD9-CM codes for comorbid conditions.

|  |  | **ICD9-CM** |
| --- | --- | --- |
| Chronic Obstructive Pulmonary Disease | | 490-496 |
| Diabetes | | 250 |
| Brain injury | | 800,801,803,850-854 |
