# Supplementary material for: Long-term exposure to air pollution and hospitalization for dementia in the Rome longitudinal study
Source: Environ Health. 2019 Aug 9;18:72. doi: 10.1186/s12940-019-0511-5 (PMC6689157; doi:10.1186/s12940-019-0511-5)
Supplement: Supplementary file 2 — Pearson correlation coefficients of exposure to air pollutants. (DOCX 14 kb) [file 12940_2019_511_MOESM2_ESM.docx]

**Additional file 2.** Pearson correlation coefficients of exposure to air pollutants.

| **Exposure** | PM_10_ | Coarse PM | PM_2.5_ | PM_25_ absorbance | NO_2_ | NOx | O_3_ |
| --- | --- | --- | --- | --- | --- | --- | --- |
| PM_10_ | 1.00 |  |  |  |  |  |  |
| Coarse PM | 0.94 | 1.00 |  |  |  |  |  |
| PM_2.5_ | 0.93 | 0.91 | 1.00 |  |  |  |  |
| PM_2.5_ absorbance | 0.61 | 0.65 | 0.65 | 1.00 |  |  |  |
| NO_2_ | 0.60 | 0.71 | 0.66 | 0.60 | 1.00 |  |  |
| NOx | 0.55 | 0.61 | 0.61 | 0.51 | 0.71 | 1.00 |  |
| O_3_ | -0.03 | -0.03 | -0.03 | -0.07 | -0.11 | -0.12 | 1.00 |
