# Supplementary material for: Long-term exposure to air pollution and hospitalization for dementia in the Rome longitudinal study
Source: Environ Health. 2019 Aug 9;18:72. doi: 10.1186/s12940-019-0511-5 (PMC6689157; doi:10.1186/s12940-019-0511-5)
Supplement: Supplementary file 3 — Association between long-term exposure to air pollution and dementia, vascular dementia, Alzheimer disease and senile dementia. Rome 2001–2013. (DOCX 16 kb) [file 12940_2019_511_MOESM3_ESM.docx]

**Additional file 3.** Association between long-term exposure to air pollution and dementia, vascular dementia, Alzheimer disease and senile dementia. Rome 2001-2013

| **Exposure** | **Dementia** | | | **Vascular dementia** | | | **Alzheimer disease** | | | **Senile dementia** | | |
| --- | --- | --- | --- | --- | --- | --- | --- | --- | --- | --- | --- | --- |
|  | **N=21,548** | | | **N=7,947** | | | **N=9,712** | | | **N=3.889** | | |
|  | **HR*** | **95%CI** | | **HR*** | **95%CI** | | **HR*** | **95%CI** | | **HR*** | **95%CI** | |
| PM_10_ *(10µg/m^3^) | 0.99 | 0.97 | 1.02 | 1.05 | 1.01 | 1.09 | 0.93 | 0.89 | 0.97 | 0.97 | 0.93 | 1.01 |
| Coarse *(5µg/m^3^) | 0.96 | 0.94 | 0.98 | 1.03 | 1.00 | 1.07 | 0.88 | 0.84 | 0.91 | 0.95 | 0.92 | 0.98 |
| PM_2.5_ *(5µg/m^3^) | 0.97 | 0.94 | 1.00 | 1.05 | 1.00 | 1.11 | 0.89 | 0.83 | 0.95 | 0.96 | 0.91 | 1.02 |
| PM_2.5_ abs *(10^-5^/m) | 0.99 | 0.96 | 1.02 | 1.14 | 1.09 | 1.18 | 0.88 | 0.83 | 0.93 | 0.92 | 0.87 | 0.97 |
| NO_2_ *(10µg/m^3^) | 0.98 | 0.97 | 1.00 | 1.07 | 1.04 | 1.09 | 0.92 | 0.90 | 0.94 | 0.97 | 0.94 | 0.99 |
| NOx *(20µg/m^3^) | 1.02 | 1.00 | 1.03 | 1.10 | 1.08 | 1.11 | 0.96 | 0.94 | 0.98 | 0.99 | 0.97 | 1.01 |
| O_3_ *(10µg/m^3^) | 1.04 | 1.01 | 1.06 | 1.00 | 0.96 | 1.04 | 0.96 | 0.93 | 1.00 | 1.17 | 1.13 | 1.21 |
| **Distance to HTR** |  |  |  |  |  |  |  |  |  |  |  |  |
| *<50* | 0.99 | 0.94 | 1.03 | 1.14 | 1.07 | 1.21 | 0.93 | 0.86 | 1.00 | 0.88 | 0.81 | 0.95 |
| *50-100* | 0.95 | 0.91 | 1.00 | 1.08 | 1.00 | 1.16 | 0.93 | 0.85 | 1.00 | 0.86 | 0.79 | 0.94 |
| *101-200* | 0.97 | 0.93 | 1.01 | 1.07 | 1.01 | 1.14 | 0.96 | 0.90 | 1.03 | 0.85 | 0.79 | 0.92 |
| *201-300* | 0.97 | 0.93 | 1.02 | 0.99 | 0.91 | 1.07 | 0.98 | 0.91 | 1.05 | 0.93 | 0.85 | 1.00 |
| *>300* | 1.00 | ref. | | 1.00 | ref. | | 1.00 | ref. | | 1.00 | ref. | |

*HR adjusted for age with baseline hazard function stratified by sex
