# Supplementary material for: Long-term exposure to air pollution and hospitalization for dementia in the Rome longitudinal study
Source: Environ Health. 2019 Aug 9;18:72. doi: 10.1186/s12940-019-0511-5 (PMC6689157; doi:10.1186/s12940-019-0511-5)
Supplement: Supplementary file 4 — Association between long-term exposure to air pollution and first hospitalization for dementia. Sensitivity analyses, Rome 2001–2013. (DOCX 16 kb) [file 12940_2019_511_MOESM4_ESM.docx]

**Additional file 4.** Association between long-term exposure to air pollution and first hospitalization for dementia. Sensitivity analyses, Rome 2001-2013

| **Exposure** | **Comorbidities adjustment** | | | **Competing risk** | | | **Narrower case definition** | | | **Primary diagnosis** | | | **Non movers** | | |
| --- | --- | --- | --- | --- | --- | --- | --- | --- | --- | --- | --- | --- | --- | --- | --- |
|  | **N=21,548** | | | **N=21,548** | | | **N=5,810** | | | **N=7,486** | | | **N=18,786** | | |
|  | **HR^1^** | **95%CI** | | **HR^2^** | **95%CI** |  | **HR^3^** | **95%CI** | | **HR^4^** | **95%CI** | | **HR^5^** | **95%CI** | |
| PM_10_ *(10µg/m^3^) | 1.00 | 0.98 | 1.03 | 1.01 | 0.99 | 1.04 | 0.95 | 0.90 | 1.00 | 0.98 | 0.93 | 1.02 | 1.00 | 0.97 | 1.03 |
| Coarse *(5µg/m^3^) | 0.98 | 0.96 | 1.00 | 1.00 | 0.98 | 1.02 | 0.95 | 0.91 | 0.99 | 0.93 | 0.90 | 0.97 | 0.98 | 0.96 | 1.00 |
| PM_2.5_ *(5µg/m^3^) | 0.99 | 0.96 | 1.03 | 1.01 | 0.97 | 1.04 | 0.95 | 0.88 | 1.02 | 0.95 | 0.89 | 1.01 | 0.98 | 0.94 | 1.02 |
| PM_2.5_ abs *(10^-5^/m) | 1.01 | 0.98 | 1.03 | 1.02 | 0.99 | 1.04 | 0.97 | 0.92 | 1.03 | 0.95 | 0.90 | 1.00 | 1.01 | 0.98 | 1.04 |
| NO_2_ *(10µg/m^3^) | 0.98 | 0.96 | 0.99 | 0.98 | 0.96 | 0.99 | 0.95 | 0.92 | 0.98 | 0.92 | 0.90 | 0.94 | 0.98 | 0.96 | 0.99 |
| NOx *(20µg/m^3^) | 1.01 | 1.00 | 1.02 | 1.01 | 1.00 | 1.02 | 0.99 | 0.97 | 1.01 | 0.95 | 0.93 | 0.97 | 1.01 | 1.00 | 1.02 |
| O_3_ *(10µg/m^3^) | 1.06 | 1.04 | 1.08 | 1.07 | 1.04 | 1.09 | 1.06 | 1.01 | 1.10 | 0.98 | 0.94 | 1.02 | 1.05 | 1.03 | 1.08 |

1 HR adjusted for age, education, place of birth, marital status, comorbidities (Chronic Obstructive Pulmonary Disease, diabetes and brain injuries), area-based socioeconomic position with baseline hazard function stratified by sex.

2 HR Models adjusted for gender, education, place of birth, marital status and area-based socioeconomic position with baseline hazard function stratified by sex, with death as competing risk.

3 HR adjusted for age, education, place of birth, marital status, area-based socioeconomic position with baseline hazard function stratified by sex. The selection of case was based on the first of at least two hospital discharges.

4 HR adjusted for age, education, place of birth, marital status, area-based socioeconomic position with baseline hazard function stratified by sex. The selection of case was based on primary diagnosis only.

5 HR adjusted for age, education, place of birth, marital status, area-based socioeconomic position with baseline hazard function stratified by sex in subject that not change residence during the follow up.
