# Supplementary material for: Long-term exposure to air pollution and hospitalization for dementia in the Rome longitudinal study
Source: Environ Health. 2019 Aug 9;18:72. doi: 10.1186/s12940-019-0511-5 (PMC6689157; doi:10.1186/s12940-019-0511-5)
Supplement: Supplementary file 8 — Previous studies on Dementia disease (overall dementia, Alzheimer’s disease and vascular dementia) and air pollution. (DOCX 16 kb) [file 12940_2019_511_MOESM8_ESM.docx]

**Additional file 8.** Previous studies on Dementia disease (overall dementia, Alzheimer’s disease and vascular dementia) and air pollution

| **Study** | **Setting** | **Period** | **Study design** | **Pollutants** | **Total/**  **controls** | **Cases** | **Type of**  **dementia** | **Outcomes** |
| --- | --- | --- | --- | --- | --- | --- | --- | --- |
| Chang et al. , 2014 | Taiwan | 1998-2010 | Cohort | NO_2_ | 29,547 | 1,720 | Overall | HR NO_2_ = 1.54 (1.34, 1.77) Q4 compared Q1 |
| Oudin et al. , 2015 | Sweden | 1993-2010 | Cohort | NOx | 1,806 | 191 for AD  111 for VD | Overall  AD  VD | HR NOx = 1.05 (0.98, 1.12) per 10 μg/m3  HR_AD_ NOx= 1.05 (0.97, 1.15) per 10 μg/m3  HR_VD_ NOx = 1.02 (0.92, 1.14) per 10 μg/m3 |
| Wu et al., 2015 | Taiwan | 2007-2010 | Case-control | PM_10,_ O_3_ | 497 | 249 for AD  125 for VD | AD  VD | OR_AD_ PM_10_ = 4.17 (2.31, 7.54) T3 compared T1  OR_VD_ PM_10_= 2.00 (1.14, 3,50) T3 compared T1  OR_AD_ O_3_ = 3.61 (1.67, 7.81) T3 compared T1  OR_VD_ O_3_ = 2.09 (1.01, 4.33) T3 compared T1 |
| Kiomourtzoglu et al. , 2015 | Northeastern USA | 1999-2010 | Cohort | PM_2.5_ | 9.8 million | 266,725 for overall  203,463 for AD | Overall  AD | HR PM_2.5_ = 1.08 (1.05, 1.11) per 1 μg/m3  HR_AD_ PM_2.5_ = 1.15 (1.11, 1.19) per 1 μg/m3 |
| Jung et al., 2015 | Taiwan | 2001-2010 | Cohort | PM_2.5_ , PM_10,_ O_3_ | 95,690 | 1,399 | AD | HR_AD_ PM_2.5_ = 1.03 (0.95, 1.11) per 13.21 g/m3  HR_AD_ O_3_ = 1.06 (1.00, 1.12) per 9.63 ppb |

Abbreviations: AD, Alzheimer’s disease; VD, vascular dementia; Q4, fourth quartile; Q1, first quartile; T3, third tertile; T1, first tertile

**Additional file 8 (Continued)**

| Chen et al., 2017a | Canada | 2001-2012 | Cohort | Distance from  high traffic road | 2,2 million | 243,611 | Overall | HR <50 m = 1,07 (1,06, 1,08) compared to >300 m |
| --- | --- | --- | --- | --- | --- | --- | --- | --- |
| Chen et al., 2017b | Canada | 2001-2013 | Cohort | PM_2.5_ , NO_2,_ O_3_ | 2,1 million | 257,816 | Overall | HR NO_2_ = 1.10 (1.08, 1.12) per 14.2 ppb  HR PM_2.5_ = 1.04 (1.03, 1.05) per 4.8 μg/m3 |

Abbreviations: AD, Alzheimer’s disease; VD, vascular dementia; Q4, fourth quartile; Q1, first quartile; T3, third tertile; T1, first tertile
